# Changes in conservation value from grasslands to savannas to forests: How a temperate canopy cover gradient affects butterfly community composition

**DOI:** 10.1371/journal.pone.0234139

**Published:** 2020-06-19

**Authors:** Ralph Grundel, Gary S. Dulin, Noel B. Pavlovic

**Affiliations:** 1 U.S. Geological Survey, Great Lakes Science Center, Chesterton, Indiana, United States of America; 2 Valparaiso University, Department of Biology, Valparaiso, Indiana, United States of America; Texas State University, UNITED STATES

## Abstract

Temperate savannas and grasslands are globally threatened. In the Midwest United States of America (USA), for example, oak savannas persist today at a small percentage of recent historic coverage. Therefore, restoration of habitats of low and intermediate canopy cover is a landscape conservation priority that often emphasizes returning tree density to a savanna-like target value. Understanding how animal species react to such changes in vegetation structure is important for assessing the value of these restoration plans. We examined how butterfly community attributes in northwest Indiana USA, including community composition, richness, and abundance responded to a grassland-to-forest gradient of canopy cover. Butterfly community composition under intermediate canopy cover differed significantly from community composition in the most open or closed-canopy habitats. Composition of the plant community in flower was a significant predictor of three assessed attributes of the butterfly community—composition, richness, and abundance. Phenology, expressed as day-of-the-year, was also a strong predictor of these butterfly community attributes. Few butterfly species were habitat specialists as adults although canopy cover was a more important predictor of adult community composition than of richness or abundance of butterflies. Therefore, adult butterfly community differences along the canopy cover gradient were less about butterfly communities filled with habitat specialists for different canopy-defined habitats and more about gradual changes in community composition along this gradient. Overall, butterfly community richness was predicted to peak at about 34% canopy cover, butterfly abundance at about 53% canopy cover, community conservation value at about 59% canopy cover, and a combination of desirable conservation attributes–high diversity, high abundance, and high conservation value–was predicted to reach a peak of co-occurrence at about 67% canopy cover suggesting that habitats of intermediate canopy cover might be particularly effective for butterfly conservation in this region.

## Introduction

Globally, temperate savannas, woodlands, and grasslands are biomes of special conservation concern [1]. Human activities have influenced the temperate forests, savannas, and grasslands of eastern North America for thousands of years [2, 3]. Nonetheless, since settlement by European-Americans, the eastern North American landscape has changed greatly in part due to conversion to agriculture and other development and due to changes in disturbance regimes, especially fire. Additionally, this landscape has experienced relatively low rates of conservation protection [1, 4]. Savanna was present on more than ten million hectares (ha) in the Midwest USA and Canada in the early nineteenth century but the rate of conversion to human use and the frequency of succession to more forested habitats, due to decreased fire frequency, have been high even compared to global trends [5–7]. Estimates of the decrease in acreage of oak dominated woodlands and savannas in this region range from 83% [8] to more than 99% [7] over the past two hundred years. Recognition of this decline has stimulated planning for large scale restoration of these habitats in the Midwest United States [8, 9].

Prairie, savanna, and forest habitats co-occur in a Midwest USA habitat transition zone with the distribution among these habitats across landscapes likely related to climatic factors, such as precipitation levels, to soil types, to landscape physiognomy, and to fire frequency [10]. In deciding habitats to prioritize during restoration along this canopy cover gradient, an understanding of how plant and animal communities are likely to change with changes in canopy cover is valuable. Previously, to provide guidance to managers as they formulate goals for restoration within this habitat mosaic landscape, we examined the effects of canopy cover on amphibians, bees, birds, and reptiles along this gradient, a group of animal taxa that differ in their vagility, size, life span, and life history habitat requirements [11–14]. Here, we add adult butterflies to this group of studied taxa for several reasons including their complex life cycles that might promote dependencies on multiple canopy conditions. Also, at the time of this study, canopy reduction to provide suitable habitat for the federally endangered Karner blue butterfly (*Lycaeides melissa samuelis*) was a primary goal for managing some of the sites we studied but the effects of this management goal on other species of butterflies or other animal or plant species was not well documented [15–17].

Concern about how changes in canopy cover and heterogeneity, due to changes in fire or cutting regimes, might affect understory communities are geographically widespread [18]. For example, canopy management via coppicing, a woodland management technique historically used in Europe but less consistently used recently, has resulted in significant changes in canopy structure in many areas of Europe [19, 20]. These changes in canopy cover have affected the abundance and distribution of a variety of plant and animal species, including arthropods such as spiders [21], beetles [22, 23], moths [24], and butterflies [25, 26]. Frequently, loss of canopy heterogeneity is associated with decline in light penetration and microclimatic heterogeneity in the understory and decline in species diversity [21]. Here we expand our investigation of how canopy cover manipulation might affect biotic diversity in the Midwest USA prairie-forest transition zone [27] by examining how canopy cover affects adult butterfly community composition, richness, and abundance. We present predictions for the response of these butterfly community attributes to canopy cover. Finally, we predict whether canopy cover might also affect the conservation value, or ability to retain threatened species, at these sites.

## Methods

We examined relationships between butterfly distribution and habitat structure along a canopy cover gradient, using an historic data set of butterfly distribution collected in 1998–1999, at 25 sites within three locations in northwest Indiana, USA. Data used for this study are publicly available at https://doi.org/10.5066/P9TYX2AG.

### Study sites

The 25 study sites were located at Indiana Dunes National Lakeshore (now National Park) (*n* = 17 sites) (41° 38’ N, 87° 09’ W) (6000 ha), Tefft Savanna Nature Preserve and Jasper-Pulaski Fish and Wildlife Area (*n* = 7 sites) (41° 10’ N, 86° 58’ W) (3250 ha), and Hoosier Prairie Nature Preserve (*n* = 1 site) (41° 31’ N, 87° 27’ W) (225 ha). Sites, and the habitat assessment methodology, are described in detail elsewhere but are reviewed here [11–13, 28]. At each site, butterflies were surveyed along either one 500-m transect, or two 250-m transects separated by 30 m to limit double counting, depending on site dimensions. Mean site area was 11.8 ha ± 2.4 (standard error, SE). Sites were situated from 0.8–80 km inland from the southern shore of Lake Michigan, averaged 1.8 km ± 0.7 (SE) between nearest neighbors, were mainly on sandy soils, and represented five replicates of each of five habitat types that may arise from historic changes to savannas. These habitat types included: (1) open habitats (< 20% canopy cover) including prairie remnants, other grasslands, and sites with low canopy cover due to past soil disturbances, such as farming and sand mining [29], (2) savannas with 20–50% oak-dominated canopy cover and little woody understory vegetation, (3) woodlands with 50–90% canopy cover and little woody understory vegetation, (4) scrub habitats with high density (> 1,000 woody stems) of black oak (*Quercus velutina*) sprouts (2.5–10 cm diameter breast height (dbh) ha^-1^ and < 5 m high) that often arise following intense fires, and (5) forests with high canopy cover (>90%) from larger trees and with several woody vegetation layers containing multiple tree species with more than 300 woody stems > 10 cm dbh ha^-1^. Dominant tree species included black oak and white oak (*Q*. *alba*) and sassafras (*Sassafras albidum*).

### Butterfly abundance determination

We surveyed butterflies 21 times (*n* = 13 in 1998 and 8 in 1999) from May 4 –October 13 in 1998 and from April 26 –October 1 in 1999. At least one genus (*Phyciodes*) contained more than one species that could not be distinguished in the field. In summing species numbers, we counted this genus as one species.

To reduce bias related to differences in detectability among butterfly species, we used distance sampling to convert raw butterfly counts to estimates of density [30, 31]. To implement distance sampling, we estimated the perpendicular distance from each observed butterfly to the transect line. If observations of a species were insufficient to estimate the detection function used in the density calculation, we pooled observations over species similar to this rarer species [31]. The distance sampling software [30] calculated effective strip width (ESW), the distance at which we expect the number of a butterfly species detected outside the ESW should equal the number of animals missed inside the ESW. Approximately 7.8% of butterflies observed could not be identified to species in the field but only to a higher taxonomic level, such as family. We apportioned those butterflies to the species level, based on the butterflies identified to species at that site daily, if possible. Density was expressed as the sum of individual species’ densities per hectare, either on a per-survey basis or the average per-survey density across all surveys.

### Predictors of butterfly abundance and distribution

We examined possible relationships between twenty-one predictors and butterfly distribution across the sites: Predictors are listed in Table 1 with brief description. Here we provide some additional information on a few of the predictors that we do not describe in detail elsewhere [11–13, 28] or for which the brief description in Table 1 might be insufficient.

**Table 1:** List of possible predictors of butterfly distribution at a northwest Indiana site.

Predictor Name	Description	Reference
*Year*	Year (factor)	
*DayofYear*	Day of year	
*Temperature*	Temperature (°C) at start of survey	
*WindSpeed*	Wind speed on Beaufort Scale (land conditions)	
*CloudCover*	Averaged cloud cover (%) measured with a densiometer in the four cardinal directions without overhead canopy cover	
*Developed*	% developed land (not in agriculture) within 800 m of the transect from 2001 National Landcover Dataset (NLCD)	[32]
*Agriculture*	% land in agriculture within 800 m of the transect from 2001 National Landcover Dataset (NLCD)	[32]
*HabitatDiversity*	Habitat within 200 m of the transect mapped into 11 classes [forests, savanna, woodland, and scrub (oak or non-oak dominated), wetlands, wetland shrub dominated, wetland forb dominated, open, human]. Based on proportion of the area with each of the habitat types, we calculated a Shannon-Weiner index of habitat diversity	[33]
*VegetationShort*	Vertical percent cover of vegetation < 0.3 m tall	[11–13, 28]
*VegetationTall*	Vertical percent cover of vegetation 0.3–1.0 m tall	[11–13, 28]
*Litter*	% litter cover	[11–13, 28]
*CanopyCover*	% canopy cover	[11]
*SEVM*	Spatial eigenvector mapping	[34, 35]
*Fire2*	total area burned over the two years preceding surveys within 200 m of the transect, divided by the total area within 200 m of the transect	[28]
*Fire15*	total area burned over the fifteen years preceding surveys within 200 m of the transect, divided by the total area within 200 m of the transect	[28]
*PlantComposition*	Principal curve ordination score describing the yearly plant community at a site	[36–38]
*FlowerComposition*	Principal curve ordination score describing the plants in flower during a survey	[37, 38]
*FlowerSpecies*	Number of species in flower, estimated by Abundance Coverage-based Estimator	[39, 40]
*FlowerStems*	Number of plant stems in flower at five counting points per survey covering 4 m^2^ of sampling area	
*AnnualsPercent*	Percent of plant stems in flower that were from annual plant species	
*NativePercent*	Percent of plant stems in flower that were from native plant species	

*SEVM*: we used an eigenvector based spatial filtering method, *SEVM* (spatial eigenvector mapping), to account for spatial trends in dependent variables in the analytical models [34, 35]. The SEVM filter helps account for the effect of spatial autocorrelation from the relationship between the environmental predictors and butterfly responses.

*PlantComposition*: in both late summer 1999 and spring/early summer 2000, we sampled vegetation twice in twenty randomly placed 1 x 2 m plots, each containing five 2:1 nested subplots. The smallest subplot in which a plant species was observed was recorded for each plot and a frequency score was assigned, with 5 representing plants found in the smallest subplot and 1 in the largest subplot and 0 when the plant species was not found in the plot [36]. We averaged the late summer and the spring/early summer scores per species across a site separately and then used the greater of the summer or spring scores to represent the maximal abundance of that plant species at that site. These maximal species abundances were then ordinated in a one-dimensional principal curve analysis [37, 38]. The ordination score characterized the plant community occurring at a site and was used as a possible predictor of the butterfly community.

*FlowerComposition*: the total number of stems (*ha*^*-1*^) with flowers within 1 m of five counting points along the 500 m transect was counted and a one-dimensional principal curve ordination score calculated to describe the flowering plants available during each cycle of observation.

*FlowerSpecies*: for the flowering plants we counted when surveying a transect, we used the daily stem counts during each of the twenty surveys to calculate an Abundance Coverage-based Estimator (ACE) estimate of richness of plants in flower across our surveys at a site [39, 40].

*FlowerStems*: was the number of stems of flowering plants counted during a survey as described for *FlowerComposition*. Total area of five points surveyed equals 4 m^2^.

Vegetation and site characteristics, including *VegetationShort*, *VegetationTall*, *Litter*, *CanopyCover* across each site were measured in six 0.05 ha (25.2 m diameter) circular plots and averaged across the site by inverse distance weighting from transect center [41].

Three responses to these environmental variables were examined: (1) *Butterfly Species Richness*–the number of butterfly species observed along a transect survey, (2) *Butterfly Composition*–the one dimensional principal curve score [38] of the butterfly community composition based on densities of the species observed, (3) *Butterfly Density*–sum of densities of individual butterfly species during the survey of a transect (# ha^-1^).

### Statistical analysis

We used PERMANOVA [42, 43] to assess the significance of differences in butterfly community composition among the five habitat types and between pairs of the habitat types. Significance of differences in abundance, richness, and conservation value across habitats was assessed by a Kruskal-Wallis nonparametric one-way analysis of variance and significance of differences between pairs of habitats by Dunn’s multiple comparisons’ test [44, 45].

Co-correspondence analysis compares ordinations of two communities sampled at the same set of sites [46, 47]. Predictive co-correspondence analysis examined whether composition of the plant community, both the overall plant community determined by sampling plots and the flowering plant community determined by counting plants in flower during the butterfly surveys, significantly predicted composition of the butterfly community.

Based on species accumulation curves [48], we estimated whether the number of species captured at a common number of individual butterfly sightings (species richness), or at a common number of sites (species density), was significantly different between pairs of habitats, based on a *z*-test [49] with *p* values adjusted for multiple tests, using the Benjamini-Hochberg procedure for multiple testing in R [50, 51]. We also calculated an Abundance Coverage-based Estimator (ACE) to estimate number of butterfly species predicted to occur across surveys at a site [39, 40].

We used boosted regression tree (BRT) analysis [52, 53] to determine how environmental predictors might be related to the three responses, *Butterfly Species* (richness), *Butterfly Composition* (community composition), and *Butterfly Density* (abundance). The implementation of BRT in the R *dismo* package [52] includes the *gbm*.*step* and *gbm*.*simplify* procedures that use cross validation to evaluate the number of regression trees and the learning rate for performing the BRT analysis. *gbm*.*simplify* then calculates a simplified model that takes into account the tradeoff between model fit and complexity [53]. The relative influence of each variable on the response was scaled to sum to 100, with higher numbers indicating stronger influence. Partial dependence plots were used to illustrate the effects of individual predictors on fitted values of the responses while averaging out the effects of the other predictors. After testing for best parameter settings for the BRT models, we used the following parameter settings in the gbm.step procedure, tree.complexity = 3, learning.rate = 0.01, bag.fraction = 0.75. We assessed the fit of the cross-validated BRT models using correlations between the predicted and observed responses first on a subset of the data that was used for training and then on the remaining data that were used to validate the model derived from the training data [52, 53]. Squaring the correlations produces a measure of how well the model results account for variation in the observed results.

We calculated nonlinear regressions [54] based on cubic polynomial or Gaussian 3-parameter peak models, choosing best fit, to produce curves describing the relationships between canopy cover and butterfly community attributes including composition, richness, abundance, and conservation value.

#### Conservation value

We calculated a butterfly community conservation value based on species’ NatureServe conservation status rank score: (1) critically imperiled, (2) imperiled, (3) vulnerable, (4) apparently secure, (5) secure [55]. Prior to weighting, we reversed the scale, so scores ranged from 1 (secure) to 5 (critically imperiled). Higher values then corresponded to more imperiled species. The conservation value index (*CVI*) was the product of the reversed NatureServe conservation status rank multiplied by a butterfly’s density, summed across butterfly species, and was calculated for each survey day for each site. We limited the calculation to those butterflies with reversed NatureServe ranks greater than 2, including only species considered vulnerable, imperiled, or critically imperiled in the index calculation.

Data collection was carried out under collection permits from the US National Park Service, Indiana Dunes National Lakeshore (now Indiana Dunes National Park) and the Indiana Department of Natural Resources, Division of Nature Preserves and endangered species permits from the US Fish and Wildlife Service. All data were collected on public lands managed by the US National Park Service or Indiana Department of Natural Resources.

## Results

Sixty-one butterfly species (counting *Phyciodes* spp. as one species) were observed among the 10,041 butterflies counted in 525 surveys. Mean densities of individual butterfly species ranged from 0.007 to 14.9 ha^-1^ when averaged across all 21 surveys at all 25 sites (Table 2). Effective strip width (ESW), ranged from 0.96 to 8.5 m (mean 3.24 m ± 0.25 SE), a nearly nine-fold difference in detectability that was used to adjust the area over which the counts were effectively made.

**Table 2:** Estimated densities (mean ± SE) ha^-1^ for 61 butterfly species observed across 25 sites in northwest Indiana, USA. Densities are mean densities from twenty-one survey dates at each site for a total of 525 surveys across sites. Habitat type with highest indicator value (Max Group) for each butterfly species [42] and Effective Strip Width (ESW) [31] for each species are shown.

Family	Common Name	Species Name	Density (mean ± SE) ha^-1^ (rank among species)	Counts	Max Group^a^^,^^b^	ESW^b^
Papilionidae	Black Swallowtail	*Papilio polyxenes*	0.115 ± 0.051 (39)	36	Open*	5.98*
	Giant Swallowtail	*Papilio cresphontes*	0.008 ± 0.006 (58)	3	Savanna	5.98*
	Eastern Tiger Swallowtail	*Papilio glaucus*	0.684 ± 0.090 (18)	215	Woodland	5.98*
	Spicebush Swallowtail	*Papilio troilus*	2.554 ± 0.465 (8)	802	Forest	5.98*
Pieridae	Checkered White	*Pontia protodice*	0.071 ± 0.046 (47)	28	Scrub	7.62*
	Cabbage White	*Pieris rapae*	1.016 ± 0.252 (14)	250	Open	4.68
	Olympia Marble	*Euchloe olympia*	0.078 ± 0.029 (46)	31	Open	7.62*
	Clouded Sulphur	*Colias philodice*	0.304 ± 0.141 (28)	100	Open***	6.26
	Orange Sulphur	*Colias eurytheme*	1.188 ± 0.500 (13)	530	Open*	8.5
	Cloudless Sulphur	*Phoebis sennae*	0.023 ± 0.016 (53)	10	Scrub	8.5
	Little Yellow	*Eurema lisa*	6.382 ± 2.789 (5)	624	Woodland	1.86
Lycaenidae	American Copper	*Lycaena phlaeas*	0.302 ± 0.252 (29)	43	Open	2.72*
	Bronze Copper	*Lycaena hyllus*	0.021 ± 0.021 (55)	3	Open	2.72*
	Coral Hairstreak	*Satyrium titus*	0.236 ± 0.128 (32)	22	Savanna	1.78*
	Edwards’ Hairstreak	*Satyrium edwardsii*	1.538 ± 0.538 (12)	146	Scrub	1.81
	Banded Hairstreak	*Satyrium calanus*	0.086 ± 0.048 (44)	8	Scrub	1.78*
	Striped Hairstreak	*Satyrium liparops*	0.011 ± 0.011 (57)	1	Open	1.78*
	Gray Hairstreak	*Strymon melinus*	0.227 ± 0.113 (34)	21	Woodland	1.78*
	Eastern Tailed Blue	*Everes comyntas*	1.879 ± 0.483 (9)	195	Open	1.97
	Spring Azure	*Celastrina ladon*	4.265 ± 1.824 (6)	470	Scrub	2.1
	Karner Blue	*Lycaeides melissa samuelis*	9.262 ± 4.949 (2)	1124	Savanna	2.31
Nymphalidae	Variegated Fritillary	*Euptoieta claudia*	0.044 ± 0.026 (51)	8	Open	3.33*
	Great Spangled Fritillary	*Speyeria cybele*	2.581 ± 0.496 (7)	559	Savanna	4.13
	Aphrodite Fritillary	*Speyeria aphrodite*	0.124 ± 0.058 (38)	22	Woodland	3.33*
	*Phyciodes* spp.	*Phyciodes spp*.	1.796 ± 0.385 (10)	249	Scrub	2.64
	Question Mark	*Polygonia interrogationis*	0.961 ± 0.168 (15)	48	Open	0.96*
	Eastern Comma	*Polygonia comma*	0.179 ± 0.070 (37)	9	Woodland	0.96*
	Mourning Cloak	*Nymphalis antiopa*	0.385 ± 0.137 (24)	19	Savanna	0.96*
	American Lady	*Vanessa virginiensis*	0.661 ± 0.153 (19)	88	Woodland	2.54
	Red Admiral	*Vanessa atalanta*	0.828 ± 0.203 (17)	57	Savanna	1.32
	Common Buckeye	*Junonia coenia*	0.374 ± 0.100 (25)	60	Open***	3.07
	Red-spotted Purple	*Limenitis arthemis astyanax*	0.210 ± 0.042 (35)	55	Scrub	4.94
	Viceroy	*Limenitis archippus*	1.700 ± 0.511 (11)	296	Open	3.32
	Northern Pearly-Eye	*Enodia anthedon*	0.007 ± 0.007 (61)	1	Forest	2.81
	Applalachian Brown	*Satyrodes appalachia*	0.580 ± 0.241 (21)	86	Savanna	2.81
	Little Wood Satyr	*Megisto cymela (includes viola)*	14.901 ± 2.621 (1)	1730	Savanna	2.21
	Common Wood-Nymph	*Cercyonis pegala*	8.097 ± 1.469 (3)	765	Scrub	1.8
	Monarch	*Danaus plexippus*	0.904 ± 0.289 (16)	341	Open	7.19
Hesperiidae	Silver-spotted Skipper	*Epargyreus clarus*	7.131 ± 1.852 (4)	408	Open	1.09
	Southern Cloudywing	*Thorybes bathyllus*	0.240 ± 0.094 (31)	29	Open	2.31*
	Northern Cloudywing	*Thorybes pylades*	0.083 ± 0.034 (45)	10	Savanna	2.31*
	Hayhurst's Scallopwing	*Staphylus hayhurstii*	0.008 ± 0.008 (58)	1	Forest	2.31*
	Dreamy Duskywing	*Erynnis icelus*	0.023 ± 0.016 (53)	3	Open	2.31*
	Sleepy Duskywing	*Erynnis brizo*	0.550 ± 0.273 (22)	57	Open	1.98
	Juvenal’s Duskywing	*Erynnis juvenalis*	0.105 ± 0.068 (40)	13	Woodland	2.31*
	Horace’s Duskywing	*Erynnis horatius*	0.340 ± 0.141 (26)	41	Woodland	2.31*
	Mottled Duskywing	*Erynnis martialis*	0.628 ± 0.582 (20)	77	Scrub	2.32
	Wild Indigo Duskywing	*Erynnis baptisiae*	0.230 ± 0.122 (33)	60	Open	4.94
	European Skipper	*Thymelicus lineola*	0.039 ± 0.023 (52)	5	Open	2.45*
	Fiery Skipper	*Hylephila phyleus*	0.049 ± 0.034 (49)	6	Scrub	2.45*
	Leonard’s Skipper	*Hesperia leonardus*	0.196 ± 0.101 (36)	25	Open	2.45*
	Peck’s Skipper	*Polites peckius*	0.089 ± 0.063 (43)	11	Open	2.45*
	Tawny-edged Skipper	*Polites themistocles*	0.070 ± 0.034 (48)	9	Open	2.45*
	Crossline Skipper	*Polites origenes*	0.447 ± 0.122 (23)	123	Scrub	5.25
	Northern Broken Dash	*Wallengrenia egeremet*	0.311 ± 0.079 (27)	40	Open	2.45*
	Delaware Skipper	*Anatrytone logan*	0.105 ± 0.051 (40)	13	Open	2.45*
	Byssus Skipper	*Problema byssus*	0.012 ± 0.012 (56)	2	Open	2.45*
	Hobomok Skipper	*Poanes hobomok*	0.259 ± 0.082 (30)	33	Savanna	2.45*
	Dun Skipper	*Euphyes vestris*	0.008 ± 0.008 (58)	1	Woodland	2.45*
	Dusted Skipper	*Atrytonopsis hianna*	0.099 ± 0.078 (42)	13	Open	2.45*
	Common Roadside-Skipper	*Amblyscirtes vialis*	0.047 ± 0.026 (50)	6	Open	2.45*

^a^ Habitat with maximum indicator value

^b^ Significance level: * *p* < 0.05, *** *p* < 0.001; For Indicator Values: proportion of randomized trials with indicator value equal to or exceeding the observed indicator value when corrected for multiple significance tests using Benjamini-Hochberg test (Benjamini and Hochberg 1995; R Core Team 2018). ESW: (Effective Strip Width) and significance.

Four of the 61 species, *Colias eurytheme*, *Colias philodice*, *Junonia coenia*, *Papilio polyxenes*, had significant indicator values for open habitats, suggesting a significant affinity for open habitats. Of the 61 species, 28 (46%) were most concentrated (highest indicator value for that habitat) in open habitats, 10 (16%) in savannas, 9 (15%) in woodlands, 11 (18%) in scrublands, and 3 (5%) in forests although only for the four species indicated were those concentrations significant.

### Relationship of butterfly community to plant community composition

**Fig 1:**
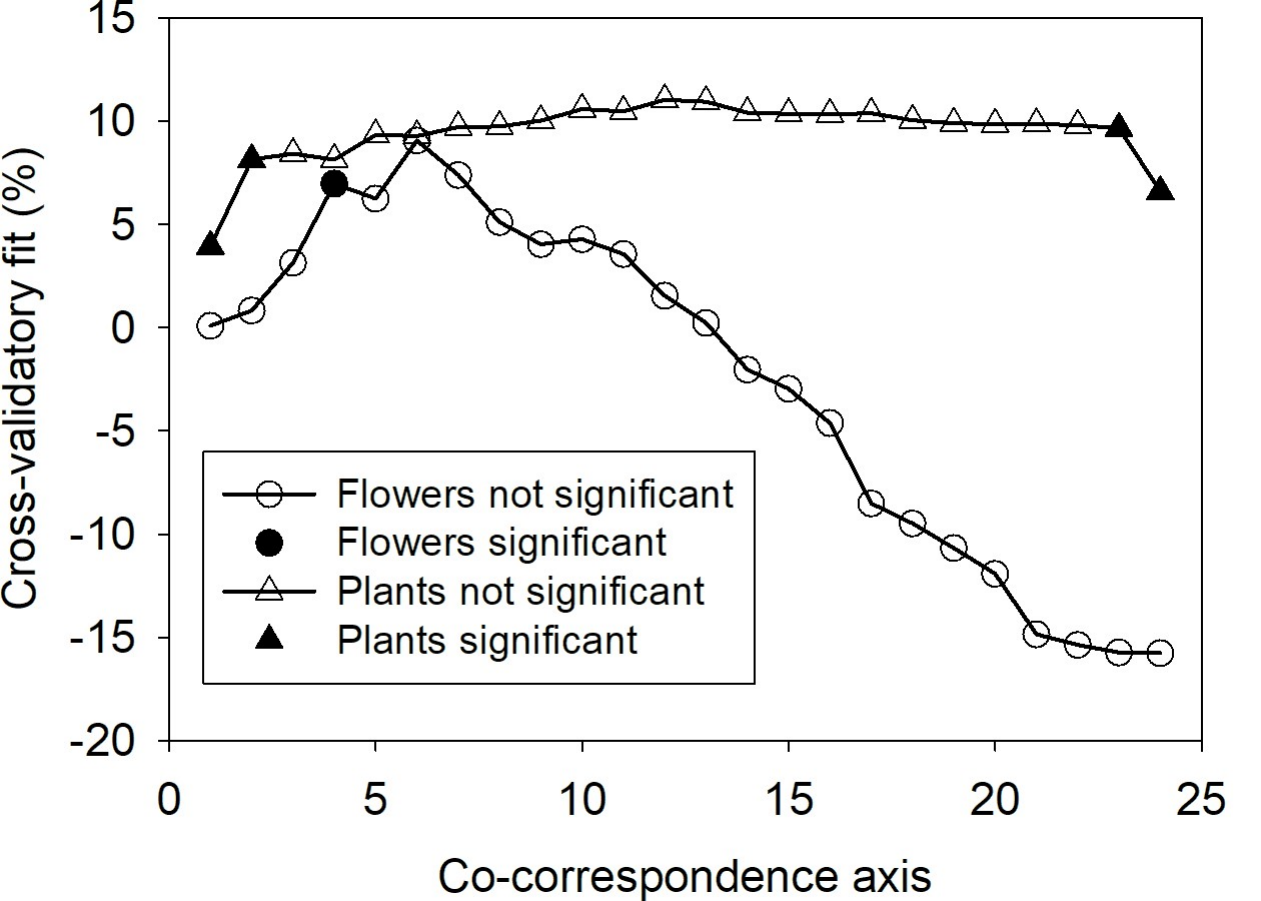
Ability of plant community composition to predict butterfly community composition determined by co-correspondence analysis. Cross-validated fit, as a function of number of ordination axes, of a predictive co-correspondence analysis of the ability of the community composition of plants in flower at time of survey (circles) and all plants present at a survey site annually (triangles) to predict the community composition of butterflies at twenty-five sites in northwest Indiana, USA. Filled circles or triangles indicate which axes are significant (*P* < 0.05) based on a permutation test [46, 47].

Both composition of the overall plant community and composition of the community of plants in flower at the time of survey were significant predictors of butterfly community composition. We performed two sets of co-correspondence analyses (Fig 1). The first analysis examined whether the plant community at a site significantly predicted the butterfly community. In this analysis, the cross-validated fit (8.1%) was nearly at a maximum with two co-correspondence analysis axes. Positive fits are typically considered significant for co-correspondence analyses in which negative fits are possible. The first two axes explained 35.4% of the variance in butterfly community composition accounted for by the 24 axes calculated. The second analysis examined whether the community of plants in flower at the time of survey significantly predicted the butterfly community. In this analysis, the cross- validated fit was nearly at a maximum with four co-correspondence analysis axes at a leave-one-out cross-validated fit of 7.0%. The first four axes explained 37.0% of the variance in butterfly community composition accounted for by the 24 axes.

### Predictors of butterfly community composition

One dimensional principal curve ordination scores were calculated to represent plant and butterfly community composition. The principal curve ordinations accounted for (a) 38.0% of the variation in flowering plant community composition (*FlowerComposition*, ln (X + 0.5) transformed) (*n* = 525 surveys), (b) 53.2% of the variation in butterfly community composition (*Butterfly Composition*, butterfly species densities (square root transformed)) (*n* = 525 surveys); (c) 58.4% of the variation in the plant community composition (*PlantComposition*) (*n* = 25 sites).

**Fig 2:**
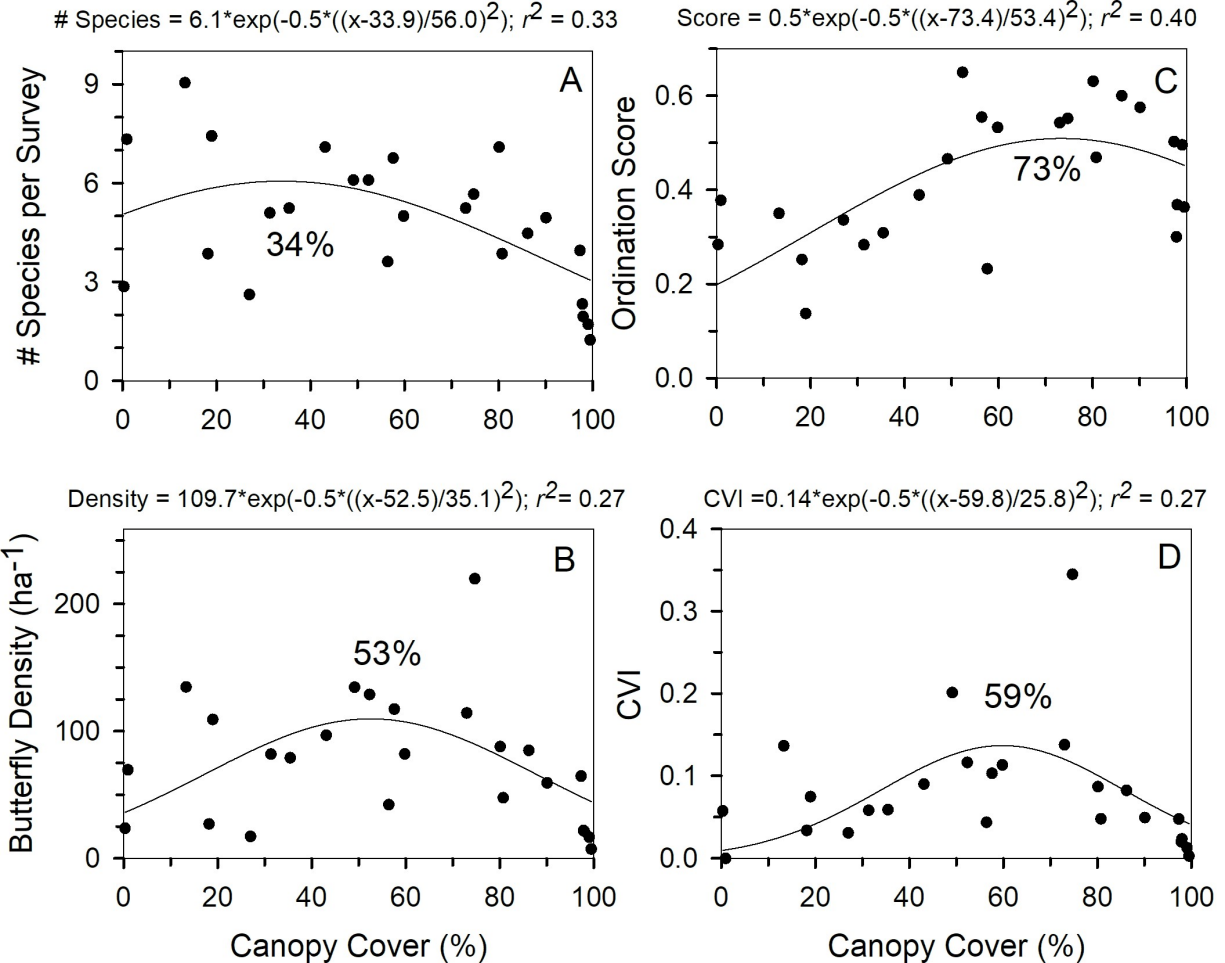
Nonlinear regression curve indicating relationship between canopy cover and butterfly community attributes. Relationship between canopy cover (%) and predicted (a) butterfly community richness, (b) density of butterflies per survey, (c) butterfly community principal curve ordination score, and (d) butterfly community Conservation Value Index (CVI). Canopy cover (%) at maximum curve Y value is shown. Based on responses averaged across 21 surveys for the 25 northwest Indiana survey sites shown.

Butterfly density peaked at about 53% canopy cover while species richness peaked at lower canopy cover, approximately 34% (Fig 2A and 2B). Per-survey density was highest in woodlands and lowest in forests (Table 3). The relationship between the butterfly community principal curve ordination score (*Butterfly Community*) and canopy cover (Fig 2C) suggests a gradual change in butterfly community composition from 0% to about 73% canopy cover at sites and then limited butterfly community compositional change at higher canopy covers. Permutational multivariate analysis of variance (PERMANOVA) indicates that these changes in composition with increased canopy cover caused butterfly community composition to differ significantly between open habitats and woodland, scrub, and forest habitats while butterfly community composition in forest habitats was significantly different than in the other four habitat types (Table 4).

**Table 3:** Mean ± SE of butterfly density (ha^-1^) and conservation value index (*CVI*) of butterfly communities in northwest Indiana by habitat type (*n* = 5 sites per habitat type). Within a column, habitats with the same superscript letter are not significantly different from each other (Kruskal-Wallis one-way analysis of variance Dunn’s nonparametric multiple comparisons’ test [44, 45, 50]).

Habitat	*Butterfly Density* (ha^-1^)	*CVI*
Open	72.9 ± 6.7^ab^	0.06 ± 0.05^ab^
Savanna	82.0 ± 9.5^ab^	0.09 ± 0.73^ab^
Woodland	106.1 ± 10.8^a^	0.11 ± 0.02^a^
Scrub	90.8 ± 13.6^b^	0.11 ± 0.13^ab^
Forest	26.4 ± 3.1^c^	0.02 ± 0.02^b^
Kruskal-Wallis χ^2^	72.1	11.7
*P*	<0.001	0.02

Values are means ± SE. Within a column, habitat values with the same superscript letter are not significantly different, Dunn’s Kruskal-Wallis multiple comparisons test [44, 45].

**Table 4:** Significance of compositional differences in northwest Indiana butterfly communities between habitat types, based on permutational multivariate analysis of variance (PERMANOVA) of butterfly densities and Sørensen distance metric [42, 43].

Comparison		*t*	*p* (adjusted)
Open	Savanna	1.30	0.34
Open	Woodland	1.55	0.05
Open	Scrub	2.06	0.04
Open	Forest	2.30	0.04
Savanna	Woodland	1.00	0.35
Savanna	Scrub	1.25	0.24
Savanna	Forest	1.72	0.04
Woodland	Scrub	0.96	0.53
Scrub	Forest	1.85	0.04

*F*_*4*,*20*_ = 2.41, *p* = 0.0002, based on randomization test with 9999 randomizations. Significance values (*p*) are adjusted for multiple tests using the Benjamini-Hochberg method [50, 51].

Canopy cover affected distribution of butterflies by family. Significantly more observations of butterflies in the family Nymphalidae occurred under canopy of trees or shrubs than was true for family Pieridae (Table 5).

**Table 5:** Percentage of individual butterfly observations in which the observed butterfly was directly below the canopy of a tree or shrub.

Family	Overall Mean	n Overall	Species Mean ± SE^1^	# Species
Papilionidae	51.0	4356	37.7 ± 13.6^ab^	3
Pieridae	15.4	1041	16.0 ± 3.7^a^	6
Lycaenidae	27.9	1579	23.5 ± 8.6^ab^	5
Nymphalidae	55.0	2025	46.3 ± 7.5^b^	12
Hesperiidae	21.9	1096	23.4 ± 3.9^ab^	10

Overall mean is based on all observations within a family. Species mean is the average percentage of observations (± SE), by species, of the # species within a family for which more than twenty observations were made.

^1^ Family species means followed by the same letter do not differ significantly from each based on Tukey multiple comparisons test [50].

**Fig 3:**
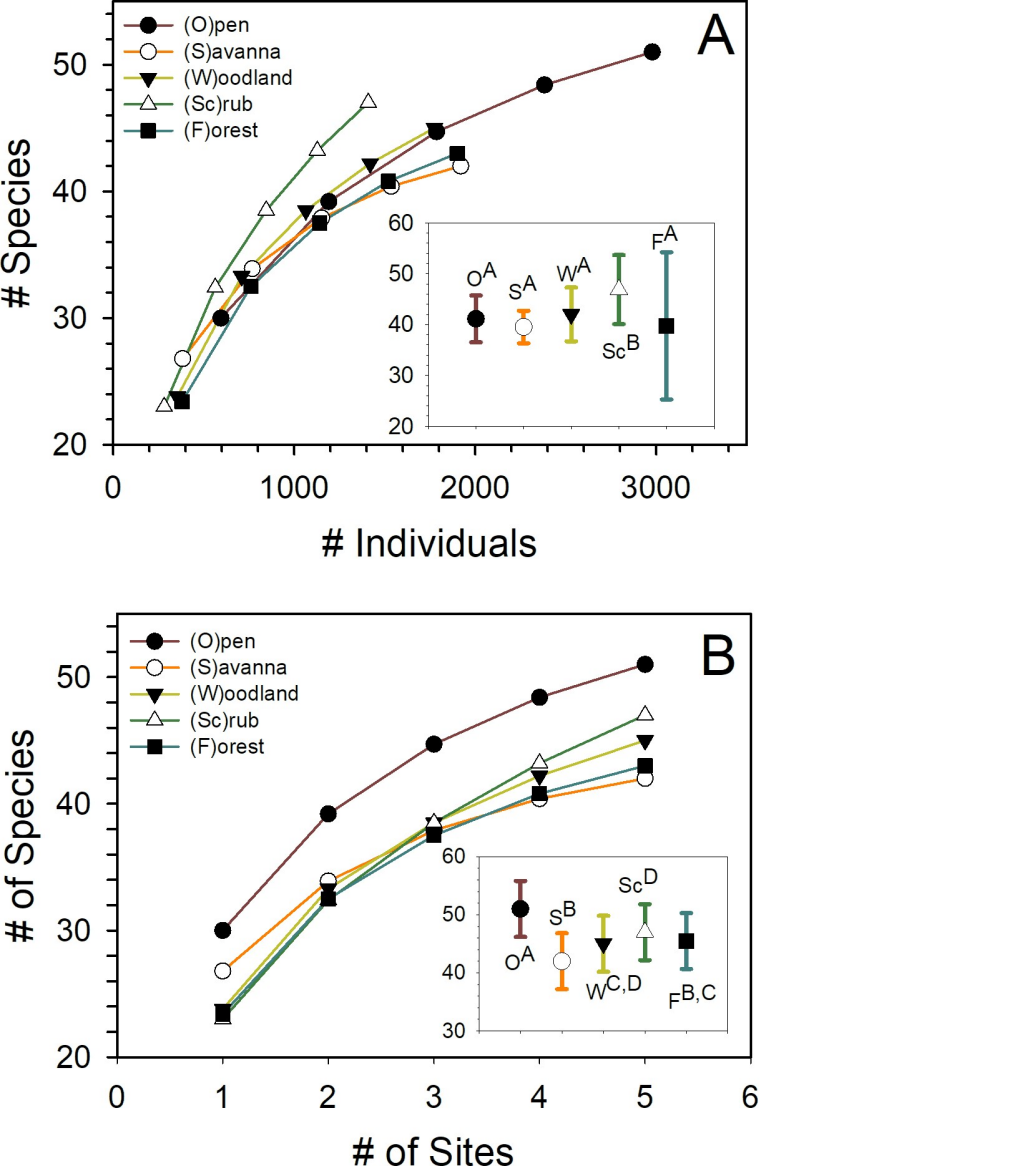
Species accumulation curves for butterflies sampled at 25 northwest Indiana sites for 21 surveys per site. Species accumulation curves as a function of (A) number of butterflies observed across sites within a habitat type, representing species richness, and (B) number of sites sampled, representing species density [48]. Inserts show mean number of species (± 95% confidence interval) accumulated at (A) a common number of individuals observed per habitat type (*n* = 1400 individuals) and (B) a common number of sites sampled per habitat type (*n* = 5 sites). Means from habitat names followed by same superscript letter do not differ significantly (*z* test with correction for multiple paired tests [49–51]). Abbreviations as shown: O–Open, S–Savanna, W–Woodland, Sc–Scrub, F–Forest.

Squared cross-validated correlations, ranged from 0.51–0.63 for the three full BRT models describing how well the twenty-one possible variables predicted the three butterfly community responses: composition, richness, and abundance (density) (Table 6). Except for butterfly species richness, simplified BRT models contained few predictors and all included day-of-the-year (*DayofYear*) and flowering plant community composition (*FlowerComposition*). In addition to those two predictors, the simplified model for butterfly community composition included canopy cover (*CanopyCover*). Twelve predictors were included in the simplified BRT model for butterfly community richness, *FlowerComposition*, *PlantComposition*, *DayofYear*, *FlowerStems*, *HabitatDiversity*, *Fire15*, *VegetationShort*, *CloudCover*, *CanopyCover*, *Agriculture*, and *SEVM* in order of importance. Although both composition of the overall plant community and the community of plants in flower were included as predictors in simplified models of at least one of the attributes, richness, composition, or abundance, of butterfly community structure, in general, the composition of plants in flower was a more important predictor of butterfly community characteristics than was the overall composition of the plant community at a site. Based on species accumulation curves (Fig 3A), butterfly species richness was higher in scrub habitats than in the other habitats and species density was highest in open habitats (Fig 3B).

**Table 6:** Relative importances for predictors of butterfly species richness, butterfly composition (principal curve score), and butterfly density as determined by boosted regression tree analysis, using procedure gbm.step [52, 53]. Importances with daggers (†) are associated with predictors that were not included in a simplified model (as determined by gbm.simplify). Ten-fold cross-validated correlation between observed and predicted response are shown at the bottom for full and simplified models as well as squared correlation for full model. Model represents predictions for individual surveys (*n* = 21 surveys per site) across 25 sites (total *n* = 525).

Predictor^1^	*Butterfly Richness*	*Butterfly Composition*	*Butterfly Density*	Mean ± Standard deviation
*Year*	3.53 †	0.47	0.68	1998.4 ± 0.5
*DayofYear*	14.18 †	46.48 †	19.78 †	199.2 ± 40.4
*Temperature*	5.70 †	2.63	5.91	26.4 ± 4.0
*WindSpeed*	0.99	1.06	1.49	1.7 ± 0.8
*CloudCover*	3.75 †	2.44	4.42	17.9 ± 18.2
*Developed*	1.62	5.03	2.76	28.8 ± 22.7
*Agriculture*	1.60 †	0.25	1.83	19.2 ± 29.6
*HabitatDiversity*	3.90†	1.36	9.59	1.3 ± 0.4
*VegetationShort*	3.97 †	2.43	1.64	60.1 ± 20.4
*VegetationTall*	0.58	1.37	10.20	59.7 ± 19.3
*Litter*	1.37	1.18	0.29	31.7 ± 19.5
*CanopyCover*	2.06 †	10.42 †	2.76	57.6 ± 32.1
*SEVM*	1.59	1.31	2.44	0 ± 4.8
*Fire2*	1.42	3.03	1.24	0.4 ± 0.5
*Fire15*	3.56 †	1.93	3.41	3.2 ± 2.0
*PlantComposition*	14.91 †	0.63	3.46	0.5 ± 0.3
*FlowerComposition*	19.58 †	8.73 †	16.38 †	0.3 ± 0.3
*FlowerSpecies*	1.29	0.37	2.56	4.8 ± 3.3
*FlowerStems*	11.75 †	2.42	7.22	184.1 ± 325.4
*AnnualsPercent*	1.81	0.98	0.85	17.5 ± 21.0
*NativePercent*	0.84	5.47	1.11	92.8 ± 18.9
Correlation ± SE full model	0.76 ± 0.02	0.79 ± 0.02	0.71 ± 0.03
Correlation ± SE simplified model	0.76 ± 0.02	0.80 ± 0.01	0.51 ± 0.04
Squared Cross-validated Correlation	0.58	0.63	0.51

^1^ *Year*, *DayofYear*, *Temperature*, *WindSpeed*, *CloudCover*, *Fire2*, *Fire15*, *FlowerComposition*, *FlowerSpecies*, and *FlowerStems* differed by survey at a given site. Remaining predictors were constant for a given site but differed among sites.

† Predictors included in simplified models.

**Fig 4:**
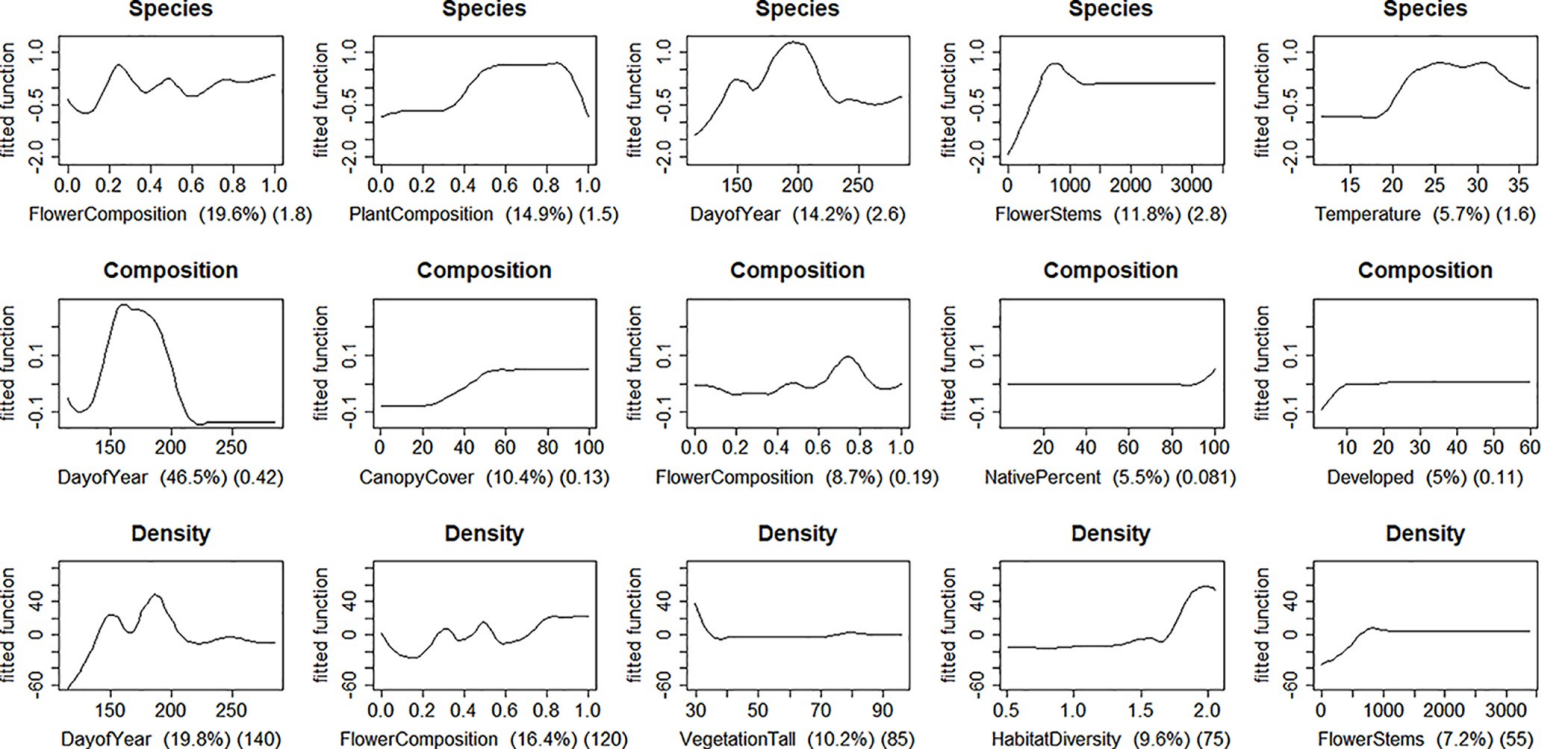
Partial dependence plots of predictor effects on three northwest Indiana butterfly community responses (*Butterfly Richness*, *Butterfly Composition*, *Butterfly Density*). Y values (*fitted function*) are the values predicted by BRT model and are centered by subtracting their mean. For each response, the five predictors accounting for the most variation, when all twenty-one possible predictors are available, are shown in descending order of relative importance. Importance values are shown in first set of parentheses on the X-axis next to the variable name and sum to 100 across all twenty-one possible predictors. The absolute range of predicted response values (maximum–minimum) for the graph is shown in the second set of parentheses. For a given graph, the effect of one predictor on one response is shown after averaging out the effects of the other predictors. Lines have been smoothed. Mean values ± SE (range) of responses (per survey): Richness: 4.82 ± 0.14 (0,14) (species survey^-1^), Composition: 0.42 ± 0.01 (0, 1), Density: 75.65 ± 4.38 (0, 801) (butterflies ha^-1^ survey^-1^). A survey is one day of observation at a site, *n* = 21 surveys per site, 25 sites.

Partial dependence plots illustrate the effect of the five predictors with the highest importance values for each butterfly community response after averaging out effects of the other predictors (Fig 4). We define effect size as the range of change in the response (maximum minus minimum) accounted for by a predictor while averaging out effects of the other predictors. For number of species observed per survey (*Richness*), effect sizes for the five predictors were between 1.42–2.82 species (range of observed values 0–14 species per survey). For community composition (*Composition*), measured as principal curve ordination value, predictor effect size was between 0.08–0.44 out of a range of possible ordination scores of 0–1. For density (*Density*), effect sizes were between 59.3–143.9 ha^-1^ (range of observed values 0–801).

As of 2017, the 61 butterfly species in Table 2 had NatureServe conservation status scores of 5 (G5) (secure) except C*elastrina ladon*, *Atrytonopsis hianna*, *Polites origenes* (G4G5), *Papilio troilus*, *Lethes appalachia* (G4), *Problema byssus* (G3G4), *Erynnis martialis* (G3), *Lycaeides melissa samuelis* (G5T2), and *Danaus plexippus* (G4T1) (NatureServe 2017). Entries with two G values (e.g., G4G5) are intermediate between the two categories. Entries with G and T indicate the global rank of overall species complex (G value) and the specific population or subspecies (T value) (e.g., G4T1 indicates the eastern North American population of *Danaus plexippus* has a status of 1 although the global population is ranked 4). The Conservation Value Index (*CVI*) was significantly different among habitats, being lowest in forests (Table 3). Nonlinear regression of conservation value versus canopy cover suggests that conservation value peaked near 59% canopy cover (Fig 2D).

**Fig 5:**
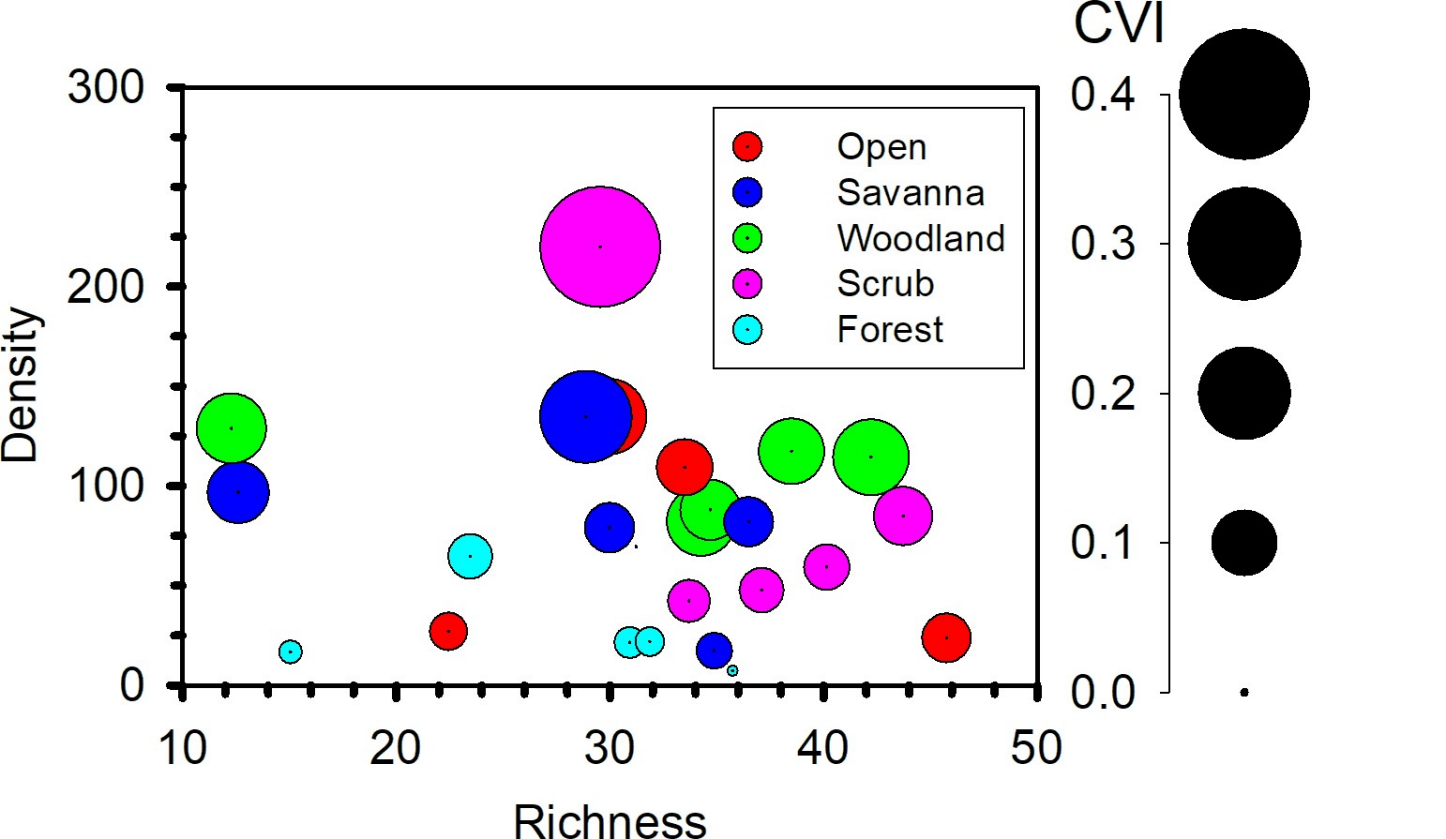
Number of butterfly species across 21 surveys (ACE) (*Richness*), mean density (ha^-1^) (*Density*), and conservation value (*CVI*) for 25 sites surveyed in northwest Indiana, USA. *CVI* is proportional to bubble area, as shown in scale at right.

**Fig 6:**
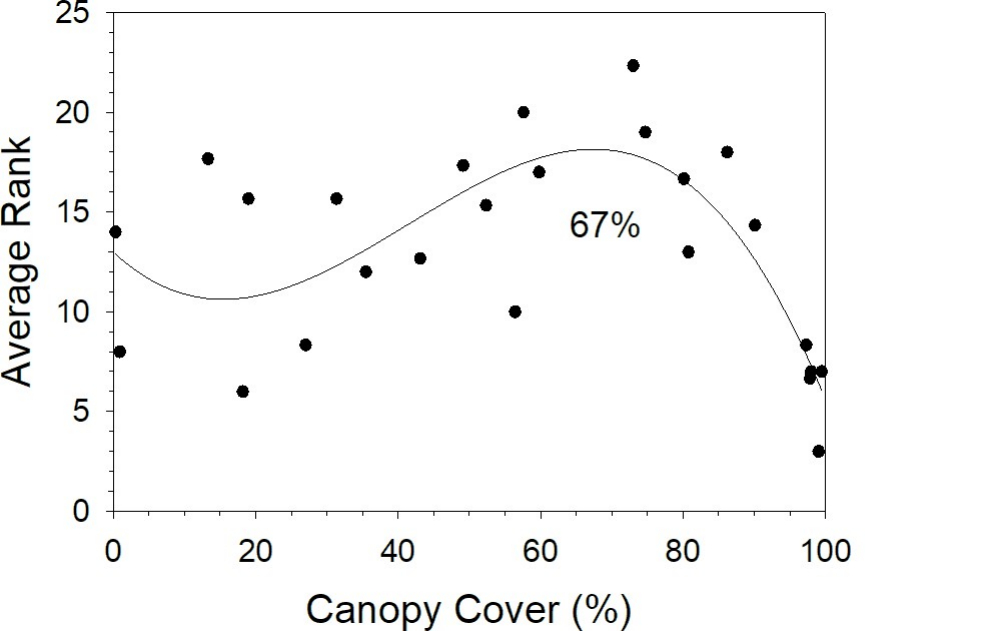
Average rank of three conservation variables versus canopy cover for 25 sites surveyed in northwest Indiana, USA. Three variables of conservation importance for the butterfly community, community richness, density, and *CVI* were ranked from 1 (lowest value of each variable, least desirable conservation outcome) to 25 (highest value, most desirable conservation outcome) and averaged across the three variables. Average of the three ranks for each site (*n* = 25 sites) is graphed against site’s mean percent canopy cover.

We examined the covariation of butterfly richness (ACE), density, and conservation value (*CVI*) per site (*n* = 25) in Fig 5. Generally, the three conservation responses were not significantly correlated with each other except for density and conservation value (Spearman rank correlation, *r*_*s*_ = 0.90, *p* < 0.001, *n* = 25, all other pairwise correlations *p* > 0.05). In Fig 5 we see that the most desirable conservation outcomes–high richness, high density, and high conservation value–did not consistently co-occur, although forested sites were not among the sites with relatively high values of each outcome. We ranked each of the 25 sites from 1 (least desirable outcome: lowest richness, density, *CVI*) to 25 (most desirable outcome: highest richness, density, *CVI*) and averaged the three ranks at each site. The averaged rank is plotted against canopy cover in Fig 6. Averaged rank increased from low to intermediate canopy cover (ca. 0–67%) and then decreased.

## Discussion

The region of the Midwest USA studied here is within an ecological transition zone where a western grassland dominated ecoregion meets an eastern hardwood forest ecoregion [27]. In this transitional landscape, a diversity of canopy covers occur and manipulation of tree density, using fire, cutting, and planting, is a common form of management [56]. This canopy diversity is likely important for maintaining resident animal and plant diversity [57]. As is true globally, interactions among climate, tree cover, and fire can affect the stability of habitat composition of a landscape that encompasses the canopy cover continuum from grasslands to savannas to forests [58–60]. In this Midwest USA region, change in fire regimes, especially fire suppression, has contributed to considerable change in canopy cover and increased local species turnover and has affected regional biodiversity. Ladwig et al. [57], for example, documented 60 years of regional change in plant community composition in a Midwest prairie-savanna-forest mosaic in Wisconsin, USA and found a near doubling of canopy cover in savannas and replacement of prairie-savanna species by forest and non-native species. However, the net effect has often not been a change in richness, but a change in composition. Maintenance of richness but with significant change in composition has been documented in many biodiversity time series globally [61, 62].

Canopy cover can affect habitat quality for adult butterflies, and subsequently affect diversity, by modifying local microclimate and light intensity [16, 21, 26, 63, 64]. Therefore, understanding responses of vegetation and animals to variation in tree density and shading are important for improving predictability of restoration outcomes and effects of landscape changes if the goal is to maintain historic species composition across heterogeneous landscapes such as the one studied here [18, 65]. Indeed, about 8% of the butterfly species from North America north of Mexico (61 of about 765) were found in the relatively small area surveyed in this study, consistent with the great range of canopy cover (~0–100%) that characterized these sites [66, 67].

Composition of the butterfly community changed gradually as canopy cover increased from about 0 to 73% cover, then exhibited less community change across higher canopy covers. The result was a forest butterfly community that was significantly different than in lower canopy cover habitats and a butterfly community in sites with the lowest canopy cover (open habitats) that was different than the communities associated with higher canopy cover (woodlands, scrublands, forests). Thus, there were different butterfly community compositions associated with low, intermediate, and high canopy cover. However, change in butterfly community composition was gradual across the canopy cover gradient and there were few butterfly species that were documented as obligates or specialists for the different habitats, open, savanna, woodland, scrub, and forest, defined by canopy cover. Only four of sixty-one butterfly species were classified as habitat specialists and these were associated with low canopy cover. Therefore, gradual compositional changes rather than abrupt turnover of habitat obligate species characterized the relationship between canopy cover and butterfly community composition.

Overall butterfly abundance (density) peaked at intermediate canopy cover, ca. 57%, and the most species (46%) were at their highest concentration in open canopy habitats while the fewest species (5%) were at their highest concentration in forest habitats with the highest canopy cover. Ten (16%) species were most concentrated in savannas, including the Karner blue butterfly, which was the subject of management emphasis at the time of this study but which has since been extirpated from the study area [17]. Richness peaked at lower canopy cover, ca. 34%. Conservation value peaked at intermediate canopy cover, ca. 58%, with the highest mean values in woodland and scrub habitats. Therefore, overall, the butterfly community exhibited gradual change in composition with increasing canopy cover resulting in low, intermediate, and high canopy cover assemblages with few habitat obligate species, overall abundance peaking at intermediate canopy cover but the most species being most concentrated in low canopy cover habitats. Overall richness, measured by species accumulation curves, was similar across habitat types but was predicted to be highest in early- to mid- successional scrub habitats. Species density, or per-survey number of species, was predicted to be highest in open habitats. These patterns of richness and abundance are similar to patterns observed elsewhere. Butterfly community richness and abundance increased in Central European woodlands [26] and Japanese forests and plantations [68] across early successional stages of forest development spanning the most open to low canopy cover stages. That pattern was true for resident, but not migrant, species in the Central European study. For all species in that study, richness and abundance declined with succession from mid-successional to forested stages, much as we documented an increase in richness and abundance from low to mid-level canopy cover and then decreases at higher canopy cover in the current study.

Patterns of variation in butterfly community composition, richness, and abundance were underlain by phenological variation. Day-of-year and composition of the community of plants in flower at the time of survey were among the most important predictors of each of those butterfly community attributes in simplified predictive models. Richness peaked in mid-season and plateaued between 500–1000 flowering stems per 4 m^2^, the total area sampled for flower abundance at each transect. Beyond patterns of seasonal phenological variation, however, butterfly community composition, richness, and abundance were best predicted by different suites of environmental attributes. In particular, the simplified model describing butterfly community richness had twelve predictors versus the two or three for composition or abundance. Beyond day-of-year and plant composition, the simplified richness model included characteristics related to habitat diversity, weather, and location, suggesting a possible biogeographic effect on richness. As noted, canopy cover was a relatively more important predictor of butterfly community composition than of richness or abundance. For example, most observations of butterflies in the families Nymphalidae and Papilionidae were of individuals under canopy while Pieridae individuals were seen under canopy infrequently. A similar pattern of distribution was observed for Papilionidae and Pieridae in Sri Lanka [69].

Various other factors were important predictors of butterfly distribution. Temperature was a moderately important predictor of richness in regression tree models, suggesting a possible survey effect, such that more butterflies were observed between 22–32°C than at higher or lower temperatures. Differences in the effective strip width of observations of different species also indicated how considering detectability affects descriptions of distribution. Specifically, we made up to nine-fold adjustments in calculations of species’ density from raw counts due to differences among species in detectability. Comparing regression tree models, percentage of native plants present was a more important predictor of butterfly community composition than of abundance or richness. Local cover of tall vegetation (0.3–1 m tall) was most important as a determinant of butterfly abundance with abundance declining beyond low cover of tall vegetation. Finally, fire is a primary management tool for habitat restoration and maintenance in the mosaic landscape of habitats studied. Short-term (2-year) and long-term (15-year) fire frequencies were of moderate to low importance as predictors of butterfly community composition, richness, or abundance. In simplified models predicting composition, richness, or abundance, only long-term fire frequency was included as a predictor, and only for butterfly richness. However, the true effect of fire frequency on the butterfly community might be through the interaction of fire and plant community composition and canopy cover. For instance, in cork oak (*Q*. *suber*) stands in Portugal, removal of understory vegetation, which is often a goal of fire management, had a strong effect on butterfly richness and abundance that peaked within three years of mechanical removal of this vegetation [70]

The butterfly community compositional differences among habitat types translated into significant differences in conservation value of the habitats for butterflies. Forests had the lowest conservation value for butterflies. Woodlands and scrublands had the highest conservation value, suggesting added conservation importance of landscapes with intermediate canopy cover and mid-successional stages—the conservation value index calculated as a measure of abundance of the most threatened butterflies in this study peaked at about 59% canopy cover. McCleery et al [18] examined how shifting woody vegetation density affected a trio of vertebrate groups (birds, bats, small mammals) in African savannas. These savannas were often undergoing changes that lead to canopy homogenization, either through loss of trees and shrubs or widespread increases in tree and shrub density. This replacement of the heterogeneous savanna canopy with more homogeneous low or high canopy conditions led to declines in diversity at the gradient extremes, illustrating a relationship between diversity of vegetation and structure and animal diversity [71, 72]. However, in the Midwest USA, doubling of canopy cover over sixty years did not result in significant change in understory plant diversity but great species turnover [57]. Košulič et al. [21] examined the relationship between spiders and canopy cover in formerly coppiced woodlands, finding that a variety of spider community attributes—richness, functional diversity, activity density, and indicators of conservation concern—were related to canopy cover but not in a manner that desirable states of those attributes co-occurred at similar canopy covers. The greatest concentration of threatened and endangered spiders was found in range of canopies covers from 65–75%, indicative of woodlands. Studies of butterflies in a regenerating forest in Sri Lanka detailed that butterfly abundance and richness declined with increasing canopy cover with richness peaking near 20% cover [69]. Similarly, Ubach et al [73] documented replacement of grassland affiliated butterfly species with closed canopy species as forests expanded and replaced grasslands in northeast Spain with grazing and farming land abandonment. The native butterfly fauna was dominated by species associated with open habitats and the replacement of those open habitats with forests was associated with frequent local extinctions of butterfly species, most of which were of butterfly species that preferred open habitats. These are all examples of disparate animal taxa responding to heterogeneity in woody vegetation cover in ways that either abundance, richness, or conservation value were positively related to canopy heterogeneity at some spatial scale or that exhibited compositional change with changes in canopy cover but not changes in richness. In the Midwest USA butterfly community studied here, composition, richness, and abundance were all related to canopy cover but not all in the same way nor with the same strength of relationship. Thus, composition was more strongly predicted by canopy cover than was richness or abundance. Richness peaked at a lower canopy cover than did overall abundance. Conservation value peaked at intermediate canopy cover. Most of the butterfly species were not significantly associated with a single habitat type along the open to forest canopy gradient examined. This suggests that multiple habitat types might be used by the adults, perhaps for different resource needs, such as for oviposition, mating, and feeding [74]. Also, we examined habitat use by adults only and not by earlier life stages. While we documented a significant relationship between habitat diversity and species richness, conservation of habitat diversity might also be important for retention of individual species since different life stages might take place predominantly in different canopy defined habitats.

Compared to results from biodiversity studies of other taxa in our study area [12, 13, 28, 75], the best predictors of different butterfly community attributes were not always the same as for those community attributes for other taxa but there were many similarities. For example, among the bird species that were significant habitat indicators most were associated either with the most open habitats, as we saw for butterflies, or the most forested habitats, which did not occur for butterflies. We found that bee abundance was negatively related to canopy cover, somewhat similar to butterflies, and positively to recent fire frequency, which was relatively an unimportant predictor of butterfly abundance [13]. Bee richness was positively related to plant richness while butterfly richness was related strongly to plant community composition but relatively weakly to plant community richness. Bee community composition was significantly related to plant richness and canopy cover, butterfly community composition was weakly predicted by plant richness and relatively strongly by canopy cover. Improving the status of savannas, and other habitats with intermediate canopy cover in the Midwest U.S., from their currently imperiled state [1, 7, 8], requires an understanding of how different taxa will respond to canopy manipulation. In studies of other taxa at the sites reported on in this study, habitats with intermediate canopy cover, such as savannas and woodlands had a high number of species relative to the canopy cover extremes, open and forest [11, 13, 28]. In this study, the species diversity patterns were somewhat different. Neither butterfly species richness nor species density peaked in savannas or woodlands. Species richness peaked in scrub habitats and species density in open habitats, suggesting that the number of butterfly species one would expect to find in a given plot on a given day was highest in open habitats but that the number of species present through time was greatest in scrub habitats. While savannas and woodlands in northwest Indiana might not be of the greatest value for maximizing the diversity of butterfly use, the value of woodlands might be found in higher use by butterfly species of conservation concern.

Conservation prioritization can be based on a variety of community attributes, such as diversity, abundance, or aiding threatened species [76, 77]. For the Midwest USA butterfly community, these desirable conservation traits–maximizing diversity, increasing abundance, aiding the most threatened species through habitat characteristics–did not necessarily co-occur in one habitat type consistently. However, co-occurrence of higher diversity, overall abundance, and abundance of threatened species did increase as canopy cover increased to intermediate levels, ca. 67%, and then declined suggesting that habitats of intermediate canopy cover might be particularly effective for butterfly conservation in this region.

## References

[1] Hoekstra JM, Boucher TM, Ricketts TH, Roberts C. Confronting a biome crisis: global disparities of habitat loss and protection. Ecol Lett. 2005;8(1):23–9. 10.1111/j.1461-0248.2004.00686.x

[2] Abrams MD, Nowacki GJ. Global change impacts on forest and fire dynamics using paleoecology and tree census data for eastern North America. Ann For Sci. 2019;76(1):8.

[3] Tulowiecki SJ, Robertson D, Larsen CPS. Oak Savannas in Western New York State, Circa 1795: Synthesizing Predictive Spatial Models and Historical Accounts to Understand Environmental and Native American Influences. Annals of the American Association of Geographers. 2019 Epub 19 August 2019. 10.1080/24694452.2019.1629871

[4] Auclair AN. Ecological factors in the development of intensive-management systems in the Midwestern United States. Ecology. 1976;57:431–44.

[5] Anderson RC, Fralish JS, Baskin JM, editors. Savannas, barrens, and rock outcrop plant communities of North America New York: Cambridge University Press; 1999.

[6] Anderson RC. Overview of midwestern oak savannas. Trans Wis Acad Sci Arts Lett. 1998;86:1–18.

[7] Nuzzo VA. Extent and status of Midwest oak savanna: presettlement and 1985. Nat Areas J. 1986;6:6–36.

[8] Fahey RT, Darling L, Anderson J. Oak ecosystems recovery plan: Sustaining oaks in the Chicago Wilderness Region. Chicago: Chicago Wilderness, 2015.

[9] Leach MK, Ross L, editors. Midwest oak ecosystems recovery plan: A call to action. Midwest Oak Savanna and Woodland Ecosystems Conference; 1995 September 27, 1995; Springfield, Missouri.

[10] Touboul JD, Staver AC, Levin SA. On the complex dynamics of savanna landscapes. Proceedings of the National Academy of Sciences. 2018;115(7):E1336–E45. 10.1073/pnas.1712356115 29378933 PMC5816152

[11] Grundel R, Pavlovic NB. Distinctiveness, use, and value of Midwestern oak savannas and woodlands as avian habitats. Auk. 2007;124(3):969–85.

[12] Grundel R, Pavlovic NB. Resource availability, matrix quality, microclimate, and spatial pattern as predictors of patch use by the Karner blue butterfly. Biol Conserv. 2007;135(1):135–44.

[13] Grundel R, Jean RP, Frohnapple KJ, Glowacki GA, Scott PE, Pavlovic NB. Floral and nesting resources, habitat structure, and fire influence bee distribution across an open-forest gradient. Ecol Appl. 2010;20(6):1678–92. 10.1890/08-1792.1 20945767

[14] Grundel R, Beamer DA, Glowacki GA, Frohnapple KJ, Pavlovic NB. Opposing responses to ecological gradients structure amphibian and reptile communities across a temperate grassland–savanna–forest landscape. Biodivers Conserv. 2014;24(5):1089–108.

[15] Grundel R, Pavlovic NB, Sulzman CL. Habitat use by the endangered Karner blue butterfly in oak woodlands: the influence of canopy cover. Biol Conserv. 1998;85:47–53.

[16] Grundel R, Pavlovic NB, Sulzman CL. The effect of canopy cover and seasonal change on host plant quality for the endangered Karner blue butterfly (*Lycaeides melissa samuelis*). Oecologia. 1998;114:243–50. 10.1007/s004420050442 28307938

[17] Patterson TA, Grundel R, Dzurisin JDK, Knutson RL, Hellmann JJ. Evidence of an extreme weather-induced phenological mismatch and a local extirpation of the endangered Karner blue butterfly. Conserv Sci Pract. 2020;2(1):e147 10.1111/csp2.147

[18] McCleery R, Monadjem A, Baiser B, Fletcher R Jr., Vickers K, Kruger L. Animal diversity declines with broad-scale homogenization of canopy cover in African savannas. Biol Conserv. 2018;226:54–62. 10.1016/j.biocon.2018.07.020

[19] Müllerová J, Szabó P, Hédl R. The rise and fall of traditional forest management in southern Moravia: A history of the past 700 years. For Ecol Manag. 2014;331:104–15. 10.1016/j.foreco.2014.07.032. PMC5435103 28529404

[20] Müllerová J, Hédl R, Szabó P. Coppice abandonment and its implications for species diversity in forest vegetation. For Ecol Manag. 2015;343:88–100. 10.1016/j.foreco.2015.02.003 28529405 PMC5435104

[21] Košulič O, Michalko R, Hula V. Impact of canopy openness on spider communities: Implications for conservation management of formerly coppiced oak forests. PLOS ONE. 2016;11(2):e0148585 10.1371/journal.pone.0148585 26845431 PMC4741389

[22] Sebek P, Altman J, Platek M, Cizek L. Is active management the key to the conservation of saproxylic biodiversity? Pollarding promotes the formation of tree hollows. PLoS ONE. 2013;8(3). 10.1371/journal.pone.0060456 23544142 PMC3609772

[23] Ramilo P, Martínez-Falcón AP, Garciá-López A, Brustel H, Galante E, Micó E. Influence of traditional management and environmental variables on Mediterranean saproxylic beetle assemblages. Environ Entomol. 2017;46(6):1235–42. 10.1093/ee/nvx140 29029177

[24] Broome A, Clarke S, Peace A, Parsons M. The effect of coppice management on moth assemblages in an English woodland. Biodivers Conserv. 2011;20(4):729–49.

[25] Benes J, Cizek O, Dovala J, Konvicka M. Intensive game keeping, coppicing and butterflies: The story of Milovicky Wood, Czech Republic. For Ecol Manag. 2006;237(1–3):353–65. 10.1016/j.foreco.2006.09.058

[26] Fartmann T, Müller C, Poniatowski D. Effects of coppicing on butterfly communities of woodlands. Biol Conserv. 2013;159:396–404. 10.1016/j.biocon.2012.11.024

[27] Transeau EN. The prairie peninsula. Ecology. 1935;16(3):423–37.

[28] Grundel R, Beamer DA, Glowacki GA, Frohnapple KJ, Pavlovic NB. Opposing responses to ecological gradients structure amphibian and reptile communities across a temperate grassland-savanna-forest landscape Biodivers Conserv. 2015;24(5):1089–108. 10.1007/s10531-014-0844-x

[29] Wilcox CA, Chun Y-M, Choi YD. Redevelopment of black oak (*Quercus velutina* Lam.) savanna in an abandoned sand mine in Indiana Dunes National Lakeshore, USA. Am Midl Nat. 2005;154:11–27.

[30] Thomas L, Laake JL, Rexstad E, Strindberg S, Marques FFC, Buckland ST, et al. Distance 6.0. Release 2. Research Unit for Wildlife Population Assessment, University of St. Andrews, UK; 2009.

[31] Buckland ST, Anderson DR, Burnham KP, Laake JL, Borchers DL, Thomas L. Introduction to Distance Sampling. Oxford, UK: Oxford University Press; 2001. 432 p.

[32] Homer CC, Huang L, Yang BW, Coan M. Development of a 2001 National Land Cover Database for the United States. Photogramm Eng Remote Sensing. 2004;70(7):829–40.

[33] Magurran AE. Measuring Biological Diversity. Oxford, U.K.: Blackwell Science; 2004.

[34] Diniz-Filho JAF, Rangel TFLVB, Bini LM. Model selection and information theory in geographical ecology. Glob Ecol Biogeogr. 2008;17(4):479–88. 10.1111/j.1466-8238.2008.00395.x

[35] De Marco P, Diniz-Filho JAF, Bini LM. Spatial analysis improves species distribution modelling during range expansion. Biol Lett. 2008;4(5):577–80. 10.1098/rsbl.2008.0210 18664417 PMC2610070

[36] Elzinga CL, Salzer DW, Willoughby JW. Measuring and monitoring plant populations. Denver, CO: Bureau of Land Management; 1998. 477 p.

[37] Walsh C. pcurve: Principal curve analysis. R package (S original by Trevor Hastie S+ library by Glenn De'ath, R port by Chris Walsh), version 0.6–3. 0.6–3 ed. Vienna, Austria: R Foundation for Statistical Computing; 2011.

[38] De'ath G. Extended dissimilarity: a method of robust estimation of ecological distances from high beta diversity data. Plant Ecol. 1999;144(2):191–9.

[39] Colwell RK. EstimateS: Statistical estimation of species richness and shared species from samples. Version 9.1. User's Guide and application published at: http://purl.oclc.org/estimates. 2013.

[40] Chao A, Chazdon RL, Colwell RK, Shen TJ. Abundance-based similarity indices and their estimation when there are unseen species in samples. Biometrics. 2006;62(2):361–71. 10.1111/j.1541-0420.2005.00489.x 16918900

[41] ESRI. ArcGIS Desktop: Release 10.4.1. Redlands, California, USA: Environmental Systems Research Institute; 2016.

[42] McCune B, Mefford MJ. PC-ORD. Multivariate Analysis of Ecological Data. Version 6.20. Version 6.04 ed: MjM Software, Gleneden Beach, Oregon, U.S.A.; 2011.

[43] Anderson MJ. A new method for non-parametric multivariate analysis of variance. Austral Ecol. 2001;26:32–46.

[44] Dunn DJ. Multiple comparisons using rank sums. Technometrics. 1964;6:241–52.

[45] Ogle D, Wheeler P, Dinno A. FSA: Fisheries Stock Analysis: R package version 0.8.26, {https://github.com/droglenc/FSA. 2019.

[46] Simpson GL. cocorresp: Co-correspondence analysis ordination methods, R package version 0.2–3. Vienna, Austria: R Foundation for Statistical Computing; 2014.

[47] ter Braak CJF, Schaffers AP. Co-correspondence analysis: a new ordination method to relate two community compositions. Ecology. 2004;85(3):834–46.

[48] Gotelli NJ, Colwell RK. Quantifying biodiversity: procedures and pitfalls in the measurement and comparison of species richness. Ecol Lett. 2001;4(4):379–91.

[49] Schenker N, Gentleman JF. On judging the significance of differences by examining the overlap between confidence intervals. American Statistician. 2001;55(3):182–6.

[50] R Core Team. R: A language and environment for statistical computing. R Foundation for Statistical Computing, Vienna, Austria v 3.5.1 URL http://www.R-project.org/. 2018.

[51] Benjamini Y, Hochberg Y. Controlling the false discovery rate: a practical and powerful approach to multiple testing. Journal of the Royal Statistical Society Series B. 1995;57:289–300.

[52] Hijmans RJ, Phillips S, Leathwick J, Elith J. dismo: Species Distribution Modeling, R package version 1.1–4, https://CRAN.R-project.org/package=dismo. R Foundation for Statistical Computing; 2017.

[53] Elith J, Leathwick J, Hastie T. A working guide to boosted regression trees. J Anim Ecol. 2008;77(4):802–13. 10.1111/j.1365-2656.2008.01390.x 18397250

[54] Systat Software Inc. SigmaPlot v 11.2. 2008.

[55] NatureServe. NatureServe Explorer: An online encyclopedia of life [web application]. Version 7.1. NatureServe, Arlington, Virginia. Available http://explorer.natureserve.org 2017 [February 21, 2018].

[56] Dey DC, Kabrick JM, Schweitzer CJ. Silviculture to restore oak savannas and woodlands. Journal of Forestry. 2017;115(3):202–11. 10.5849/jof.15-152

[57] Ladwig LM, Damschen EI, Rogers DA. Sixty years of community change in the prairie-savanna-forest mosaic of Wisconsin. Ecol Evol. 2018;8:8458–66. 10.1002/ece3.4251 30250715 PMC6145032

[58] Hirota M, Holmgren M, Van Nes EH, Scheffer M. Global Resilience of Tropical Forest and Savanna to Critical Transitions. Science. 2011;334(6053):232–5. 10.1126/science.1210657 21998390

[59] Staver AC, Archibald S, Levin SA. The global extent and determinants of savanna and forest as alternative biome states. Science. 2011;334(6053):230–2. 10.1126/science.1210465 21998389

[60] Buisson E, Le Stradic S, Silveira FA, Durigan G, Overbeck GE, Fidelis A, et al Resilience and restoration of tropical and subtropical grasslands, savannas, and grassy woodlands. Biological Reviews. 2018. 10.1111/brv.12470 30251329

[61] Blowes SA, Supp SR, Antão LH, Bates A, Bruelheide H, Chase JM, et al The geography of biodiversity change in marine and terrestrial assemblages. Science. 2019;366(6463):339–45. 10.1126/science.aaw1620 31624208

[62] Finderup Nielsen T, Sand-Jensen K, Dornelas M, Bruun HH. More is less: net gain in species richness, but biotic homogenization over 140 years. Ecol Lett. 2019;22(10):1650–7. 10.1111/ele.13361 31364805

[63] Stuhldreher G, Fartmann T. Threatened grassland butterflies as indicators of microclimatic niches along an elevational gradient–Implications for conservation in times of climate change. Ecol Indicators. 2018;94:83–98. 10.1016/j.ecolind.2018.06.043.

[64] Kleckova I, Konvicka M, Klecka J. Thermoregulation and microhabitat use in mountain butterflies of the genus *Erebia*: Importance of fine-scale habitat heterogeneity. J Therm Biol. 2014;41(1):50–8. 24679972 10.1016/j.jtherbio.2014.02.002

[65] Brudvig LA. Toward prediction in the restoration of biodiversity. J Appl Ecol. 2017;54(4):1013–7. 10.1111/1365-2664.12940

[66] Miller LD, Brown FM. A catalogue/checklist of the butterflies of America, north of Mexico: J Lepid Soc; 1981.

[67] Ferris CD. A Catalogue/Checklist of the Butterflies of America North of Mexico. The J Lepid Soc. 1989;Memoir No.3:103.

[68] Ohwaki A, Maeda S, Kitahara M, Nakano T. Associations between canopy openness, butterfl y resources, butterfl y richness and abundance along forest trails in planted and natural forests. Eur J Entomol. 2017;114:533–45.

[69] Weerakoon B, Bandara A, Ranawana K. Impact of canopy cover on butterfly abundance and diversity in intermediate zone forest of Sri Lanka. Journal of Tropical Forestry and Environment. 2015;5(1):41–6.

[70] Verdasca MJ, Leitão AS, Santana J, Porto M, Dias S, Beja P. Forest fuel management as a conservation tool for early successional species under agricultural abandonment: The case of Mediterranean butterflies. Biol Conserv. 2012;146(1):14–23. 10.1016/j.biocon.2011.10.031

[71] Lawler JJ, Ackerly DD, Albano CM, Anderson MG, Dobrowski SZ, Gill JL, et al The theory behind, and the challenges of, conserving nature's stage in a time of rapid change. Conserv Biol. 2015;29(3):618–29. 10.1111/cobi.12505 25922899

[72] Huston M. A General Hypothesis of Species Diversity. The American Naturalist. 1979;113(1):81–101. 10.1086/283366

[73] Ubach A, Páramo F, Gutiérrez C, Stefanescu C. Vegetation encroachment drives changes in the composition of butterfly assemblages and species loss in Mediterranean ecosystems. Insect Conserv Divers. 2020;13(2):151–61. 10.1111/icad.12397

[74] Wiklund C. Oviposition, feeding and spatial separation of breeding and foraging habitats in a population of *Leptidea sinapis* (Lepidoptera). Oikos. 1977;28:56–68.

[75] Grundel R, Pavlovic NB. Response of bird species densities to habitat structure and fire history along a Midwestern open-forest gradient. Condor. 2007;109:734–49.

[76] Grundel R, Pavlovic NB. Using conservation value to assess land restoration and management alternatives across a degraded oak savanna landscape. J Appl Ecol. 2008;45(1):315–24. 10.1111/j.1365-2664.2007.01422.x

[77] Capmourteres V, Anand M. “Conservation value”: a review of the concept and its quantification. Ecosphere. 2016;7(10):e01476 10.1002/ecs2.1476

